# Supplementary material for: From colonisation to chronicity: adaptation of Mycobacterium abscessus in the cystic fibrosis lung environment
Source: Microbiology (Reading). 2026 Mar 24;172(3):001677. doi: 10.1099/mic.0.001677 (PMC13012788; doi:10.1099/mic.0.001677)
Supplement: Uncited Table S1. [file mic-172-01677-s001.pdf]

From colonisation to chronicity: adaptation of *Mycobacterium abscessus* in the cystic fibrosis lung environment

Niamh Duggan[1,2], Deirdre Keating[1,2,3], Joanna Drabinska[1,2], Ciarán J. Carey[1,2], Guerrino Macori[4], Kirsten Schaffer[1,3], Siobhán McClean[1,2*].

Supplementary data

Table S1: Isolates selected for the construction of the phylogenetic tree and DCC clustering. Taken from (24)

Sample	Subspecies	Cluster	Patient	Origin	Continent	Country	Location	Sample Date	Reference
ERR3198402	*M. a. abscessus*	DCC1	GOSH18	CF	Europe	UK	London_GOSH	11/03/2008	(88)
ERR4020109	*M. a. abscessus*	DCC1	Ireland_12	CF	Europe	UK	Ireland_A	Not present	(89)
ERR459974	*M. a. abscessus*	DCC1	SVH_S	CF	Europe	UK	Dublin	Not present	(21)
ERR4020060	*M. a. abscessus*	DCC2	Ireland_11	CF	Europe	UK	Ireland_E	Not present	(89)
ERR4020088	*M. a. abscessus*	DCC2	Ireland_14	CF	Europe	UK	Ireland_A	Not present	(89)
ERR363330	*M. a. abscessus*	DCC2	AHL_C	CF	Europe	UK	Alder_Hey	28/11/2011	(21)
ERR4020112	*M. a. abscessus*	DCC4	Ireland_10	CF	Europe	UK	Ireland_A	Not present	(89)
ERR340552	*M. a. abscessus*	DCC4	RBL_Q	CF	Europe	UK	London_Brompton	Not present	(21)
ERR459969	*M. a. abscessus*	DCC4	SVH_I	CF	Europe	UK	Dublin	Not present	(21)
SRR7800718	*M. a. abscessus*	DCC5	CF00039	CF	North America	USA	TX	27/06/2015	(90)
ERR369139	*M. a. abscessus*	DCC5	DEN_Q	CF	Europe	Denmark	Copenhagen	Not present	(21)
ERR3198504	*M. a. abscessus*	DCC5	GOSH8	CF	Europe	UK	London_GOSH	29/06/2007	(88)
ERR3198392	*M. a. abscessus*	DCC5	HVH43	Non-CF	Europe	Spain	Barcelona_HVH	23/02/2013	(88)
ERR494976	*M. a. abscessus*	DCC5	SWE_B	CF	Europe	Sweden	Sweden	Not present	(21)
ERR363431	*M. a. abscessus*	DCC5	UNC_BK	CF	North America	USA	UNC_Chapel_Hill	23/05/2008	(21)
ERR373960	*M. a. abscessus*	DCC5	UNC_CU	CF	North America	USA	UNC_Chapel_Hill	20/10/2005	(21)
ERR373956	*M. a. abscessus*	DCC5	UNC_K	CF	North America	USA	UNC_Chapel_Hill	07/07/2005	(21)

Table S2: Presence of genes associated with antimicrobial resistance.

Antimicrobial class	Antibiotic	Patient isolate					
**ß-lactamases**	Cephalosporins, penems, penams	4.1	4.2	7.1	7.2	13.1	13.6
**Rifamycin**		4.1	4.2	7.1	7.2	13.1	13.6
**Glycopeptides**		4.1	4.2	7.1	7.2	13.1	13.6
**Aminoglycosides**	Kanamycin	4.1	4.2	7.1	7.2		13.6
**Aminoglycosides**	Kanamycin A					13.1	13.6
**Aminoglycosides**	Neomycin						13.6 (2 mutations)
**Aminoglycosides**	Gentamycin						13.6
**Aminoglycosides**	Gentamycin C						13.6
**Aminoglycosides**	Tobramycin						13.6 (3 mutations)
**Aminoglycosides**	Amikacin						13.6 (2 mutations)
**Macrolides**	Clarithromycin	4.1	4.2	7.1	7.2	13.1	13.6
**Disinfecting agents/antiseptics**				7.2	7.2		

Table S3: Proteins significantly decreased in abundance by ≥ 1.5-fold in late infection isolates

	M. tb gene ID	*M. tb* protein name	Fold change	Name	Tuberculist classification	p-value	UP[a]	MW[b]	SC[c]
**P4**	**ChsH3**	Q6MWW2	Unique	Possible 2-enoyl acyl-coA hydratase	Intermediary metabolism and respiration	0.01657	2	7.29	36.20
**P7**	**FbiC**	P9WP77	1.55	Probable F420 biosynthesis protein	Intermediary metabolism and respiration	0.02329	9	77.23	19.10
	**Rv2169c**	O53503	1.77	Probable conserved transmembrane protein	Cell wall processes	0.02500	4	20.37	21.30
	**mce2B, Rv0590**	O07788	2.05	Mce-family protein	Virulence, detoxification, adaptation	0.00288	2	38.26	7.90
	**cysNC, cysN**	P9WNM5	2.18	Probable bifunctional enzyme cysn/cysc: sulfate adenyltransferase (subunit 1) + adenylylsulfate kinase	Intermediary metabolism and respiration	0.02690	5	18.24	48.60
	**recR, Rv3715c**	P9WHI3	2.34	Probable recombination protein	Information pahways	0.01228	40.9	23.33	40.90
	**Mce3A,**	L7N698	2.40	Mce-family protein mce3a	Virulence, detoxification, adaptation	0.01339	14	48.06	49.30
	**Rv1697**	O33198	2.88	Conserved hypothetical protein	Conserved hypothetical	0.00519	8	39.92	30.00
	**Mce3B,**	O53968	3.33	Mce-family protein mce3b	Virulence, detoxification, adaptation	0.00013	5	23.16	32.40
	**Rv3277**	P96882	4.19	Probable conserved transmembrane protein	Cell wall processes	0.01718	2	27.04	8.70
	**YrbE4A,**	O53546	4.38	Conserved hypothetical integral membrane protein yrbe4a. Possible ABC transporter.	Virulence, detoxification, adaptation	0.00890	4	30.05	13.00
	**Mce1C**	P9WP77	5.16	Mce-family protein mce1c	Virulence, detoxification, adaptation	0.00463	5	38.12	22.10
	**MprB**	P9WGL1	Unique	Two component sensor kinase MprB	Regulatory proteins	0.00060	2	35.30	4.90
	**Rv3677c**	I6XHY3	Unique	Possible hydrolase	Intermediary metabolism and respiration	0.00029	5	27.52	36.00

[a]*Unique peptides: The number of peptide sequences unique to a protein;* [b]*MW: Molecular weight as determined by TIMS-ToF LC-MS using M. abscessus* strain ATCC 19977 *database;* [C]*SC: % sequence covered by matching peptides for the identified protein in the database*
